# Haplo2D6: a web-based tool for automated *CYP2D6* haplotype and phenotype inference

**DOI:** 10.1093/bioadv/vbag136

**Published:** 2026-05-11

**Authors:** Maria Carolina Silva de Barros Puça, Diego Mariano, Yanka Evellyn Alves Rodrigues Salazar, Raquel Cardoso de Melo-Minardi, Tais Nobrega de Sousa

**Affiliations:** Molecular Biology and Malaria Immunology Research Group, Instituto René Rachou, Fiocruz Minas, Fundação Oswaldo Cruz, Fiocruz, Belo Horizonte, 30190-002, Brazil; Department of Microbiology, Tumor and Cell Biology, Karolinska Institutet, Solna, 171 65, Sweden; Laboratory of Bioinformatics and Systems (LBS), Department of Computer Science, Universidade Federal de Minas Gerais, Belo Horizonte, 31270-901, Brazil; Molecular Biology and Malaria Immunology Research Group, Instituto René Rachou, Fiocruz Minas, Fundação Oswaldo Cruz, Fiocruz, Belo Horizonte, 30190-002, Brazil; Department of Microbiology, Tumor and Cell Biology, Karolinska Institutet, Solna, 171 65, Sweden; Laboratory of Bioinformatics and Systems (LBS), Department of Computer Science, Universidade Federal de Minas Gerais, Belo Horizonte, 31270-901, Brazil; Molecular Biology and Malaria Immunology Research Group, Instituto René Rachou, Fiocruz Minas, Fundação Oswaldo Cruz, Fiocruz, Belo Horizonte, 30190-002, Brazil; Department of Microbiology, Tumor and Cell Biology, Karolinska Institutet, Solna, 171 65, Sweden

## Abstract

**Motivation:**

Haplo2D6 is a free, browser-based tool that automates the translation of *CYP2D6* genotype data into metabolizer phenotypes. Using haplotype reconstruction with the PHASE algorithm, integration of copy number variation estimates, and curated definitions from ClinPGx and PharmVar, Haplo2D6 assigns star alleles, incorporates gene deletions and duplications, calculates activity scores (AS), and predicts phenotype.

**Results:**

This standardized, high-throughput approach improves reproducibility and reduces interpretation errors compared to manual workflows. It runs in any modern web browser, requires no installation, supports batch processing, and generates downloadable, human-readable results.

**Availability and implementation:**

Haplo2D6 is accessible at https://bioinfo.dcc.ufmg.br/Haplo2D6/ without registration.

## 1 Introduction

Cytochrome P450 2D6 (CYP2D6) is one of the most polymorphic and clinically relevant genes in human pharmacogenetics, responsible for metabolizing approximately 20–25% of clinically prescribed drugs, including antidepressants, antipsychotics, beta-blockers, opioids, chemotherapeutics, and the antimalarial primaquine (Gaedigk *et al.* 2008, Hicks *et al.* 2013, St Sauver *et al.* 2017, Saravanakumar *et al.* 2019). Its genetic diversity is shaped by single-nucleotide variants (SNVs), insertions/deletions (indels), and structural variation, including copy number variations (CNVs) and complex rearrangements such as hybrid genes and gene conversions involving the CYP2D7 pseudogene (Gaedigk *et al.* 2008, Gaedigk 2013, Gaedigk *et al.* 2017). This variability leads to considerable inter-individual differences in drug response, impacting both therapeutic efficacy and the risk of adverse reactions (Hicks *et al.* 2013, Baird *et al.* 2018, Caudle *et al.* 2020).

Phenotypes resulting from *CYP2D6* genetic variation range from poor metabolizers (PM) to ultrarapid metabolizers (UM), with intermediate (IM) and normal metabolizers (NM) in between (Gaedigk *et al.* 2018, Caudle *et al.* 2020). At the extremes, altered metabolism can result in therapeutic failure or drug toxicity, particularly for medications requiring bioactivation (St Sauver *et al.* 2017, Caudle *et al.* 2020, Silvino *et al.* 2020). Accurately predicting a patient’s phenotype requires mapping genetic variants to star alleles and calculating activity scores according to guidelines from the Clinical Pharmacogenetics Implementation Consortium (CPIC) (https://cpicpgx.org/gene/cyp2d6/) (Hicks *et al.* 2013, Gaedigk *et al.* 2018, Caudle *et al.* 2020).

Manual interpretation of genotypes is labour-intensive, error-prone, and challenging for large datasets. Existing tools, such as the HaploStats R package and phasing algorithms like PHASE, while effective, often require command-line expertise or programming skills, complex installations, and may lack scalability for large-scale studies involving thousands of individuals. In addition, several star allele calling tools such as Aldy, PyPGx, StellarPGx, and PAnno have been developed, many of which require command-line environments or local installation and are primarily designed to process sequencing-derived variant files (e.g. VCF). Recognizing these challenges, we developed Haplo2D6, a web-based application that automates the complete process of *CYP2D6* haplotype and phenotype inference in a reproducible and accessible manner. Haplo2D6 is particularly suited for genotype data generated from targeted assays (e.g. qPCR-based methods), which are widely used and do not require advanced bioinformatics expertise, thereby enabling broader accessibility for pharmacogenetic analysis.

## 2 Methods

**Figure 1:**
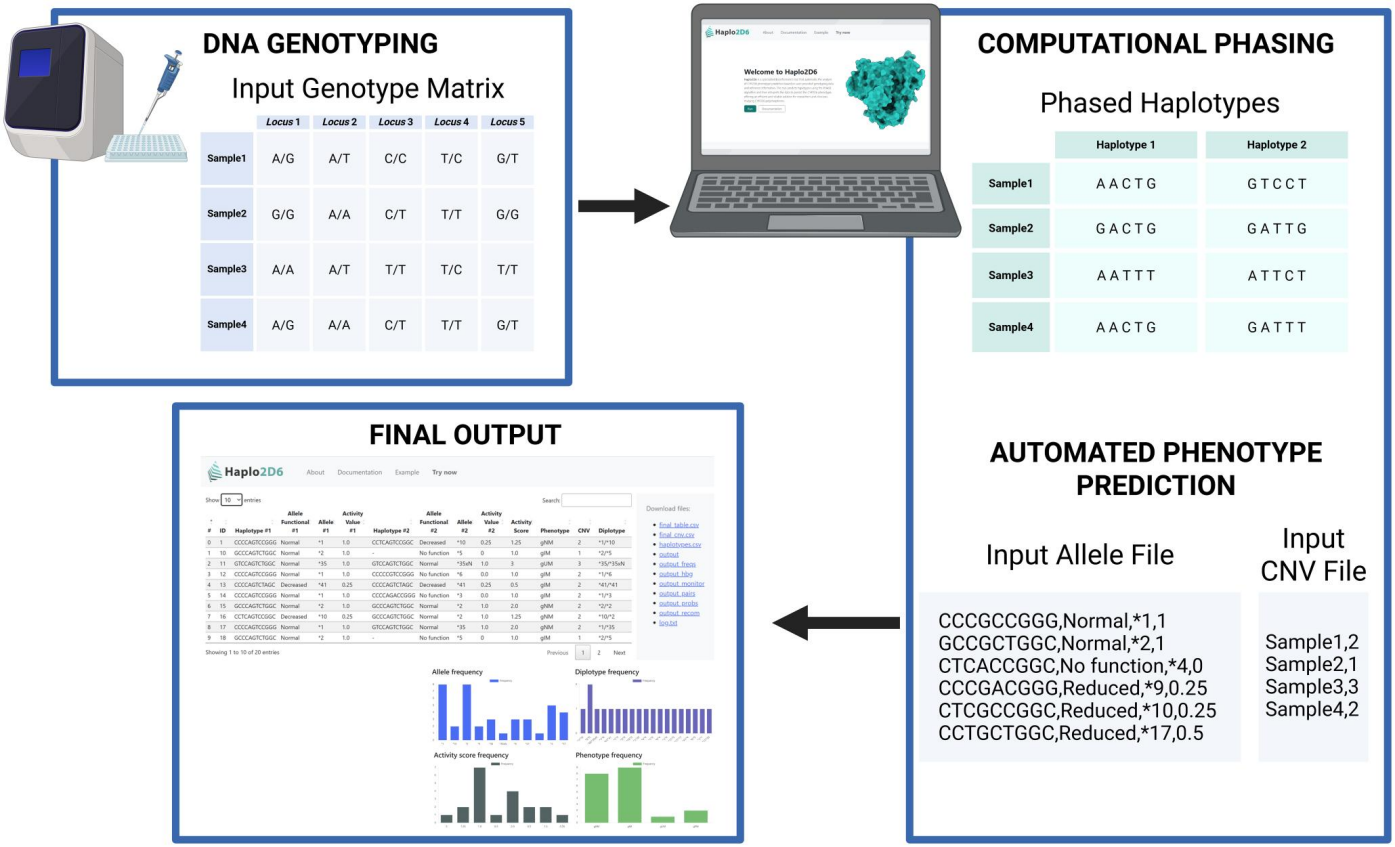
Haplo2D6 workflow for automated CYP2D6 phenotype prediction. Haplo2D6 analysis involves three main steps: (i) preparing genotype data and the allele reference file, (ii) reconstructing haplotypes using PHASE with integrated copy number variation (CNV) estimates, and (iii) assigning star alleles, calculating activity scores (AS), and predicting phenotype. Created in BioRender.

Haplo2D6 was designed to streamline pharmacogenetic workflows, providing researchers and clinicians with a reliable and user-friendly platform (Fig. 1). The process begins with the submission of *CYP2D6* genotype data in tabular format, along with an accompanying allele reference file provided by the user. This allele reference file, in CSV, must map genetic variants to *CYP2D6* star alleles, specify their function (Normal, Decreased or No function), indicate the star allele designation (e.g. *1, *2, *4), and include the allele activity value. The variant positions in the allele reference file must follow the same order as in the genotype file. Source material for CYP2D6 phenotype prediction can be found on PharmGKB (https://www.pharmgkb.org/page/cyp2d6RefMaterials) and on PharmVar (https://www.pharmvar.org/gene/CYP2D6).

Haplotype inference is a crucial step in the Haplo2D6 workflow. Haplo2D6 integrates PHASE (version 2.1.1), a Bayesian statistical method for haplotype reconstruction (Stephens *et al.* 2001, Stephens and Donnelly 2003). PHASE uses probabilistic modelling to infer the most likely arrangement of alleles across the two chromosome copies (haplotypes) for each individual, accounting for linkage disequilibrium patterns and allele frequencies estimated directly from the dataset. Phasing is performed jointly across all individuals without an external reference panel. It is important to note that the accuracy of haplotype inference using PHASE depends on dataset characteristics such as sample size, number of loci, allele frequencies, and linkage disequilibrium patterns, as the method relies on population-level information for inference. As a result, performance may be reduced in small or less informative datasets, and results should be interpreted with caution.

Once haplotypes are reconstructed, Haplo2D6 compares each haplotype sequence against the user-provided allele reference file to identify the corresponding *CYP2D6* star alleles. This precise haplotype-based matching ensures accurate allele assignment. From there, the tool assigns the activity value for each star allele, sums them to obtain the activity score (AS) per individual, and assigns the corresponding metabolizer phenotype (PM, IM, NM) according to CPIC guidelines (Caudle *et al.* 2020).

The tool generates comprehensive reports that map each variant to its respective star allele, document the diplotype and activity score, and provide a clear phenotype classification. These outputs are available for download as CSV files, facilitating integration with downstream statistical analyses and clinical workflows.

Haplo2D6 incorporates user-provided copy number variation (CNV) information during the analysis. CNV data can be included through the “Set parameters (advanced)” option, where users may specify the number of gene copies for each individual. This information can be provided in a simple tabular format using either comma- or tab-separated values (e.g. ID, CNV). By default, two copies are assumed. In cases of gene deletion, one star allele is replaced with *5, indicating the absence of the gene, thereby reducing the total activity score and potentially altering the metabolizer classification. Conversely, in cases of gene duplication or multiplication (e.g. *1x2, *1x ≥ 3), Haplo2D6 incorporates the increased copy number into the activity score calculation, which may raise the total activity score and shift the predicted phenotype from a normal metabolizer to an ultrarapid metabolizer. When identical alleles are present, the duplicated allele can be inferred; however, when different alleles are present, it is not possible to determine which allele is duplicated, and the result is reported as indeterminate.

## 3 Results

### 3.1 Implementation and accessibility

Haplo2D6 operates entirely within a web browser, eliminating the need for installation or registration. It is compatible with all major operating systems and supports both single-sample and batch processing modes. The interface is intuitive, enabling users without bioinformatics expertise to perform complex haplotype and phenotype inference in minutes. Regular updates ensure that the platform reflects the latest data from PharmVar and CPIC.

To demonstrate the utility of Haplo2D6, we analyzed *CYP2D6* genotype data from a cohort of 1300 patients from a malaria endemic area (Puça *et al.* 2025). The cohort was genotyped using a panel of 12 *CYP2D6* variants, allowing the identification of common alleles including *1, *2, *3, *4, *5, *6, *9, *10, *17, *29, *34, *35, *39, and *41. The genotype data were processed in batch mode, enabling automated haplotype assignment, activity score calculation, and phenotype prediction. The analysis revealed a distribution of metabolizer phenotypes consistent with prior population studies in the region (Brasil *et al.* 2018, Ladeia-Andrade *et al.* 2019, Silvino *et al.* 2020), highlighting the platform’s ability to generate reliable results efficiently. Haplo2D6 offers several distinct advantages: ease of use without the need for command-line operations, platform independence, up-to-date allele definitions, support for large-scale batch processing, and transparent algorithms documented for reproducibility.

## 4 Discussion

Haplo2D6 addresses critical barriers to implementing pharmacogenetics in both research and clinical practice by providing an accessible, standardized, and accurate framework for *CYP2D6* haplotype and phenotype inference from genotype data derived from targeted assays. By automating haplotype reconstruction, star allele assignment, and phenotype prediction, the platform reduces reliance on complex bioinformatics workflows and improves reproducibility. The current implementation also supports the integration of user-provided copy number variation (CNV) information, enabling CNV-aware phenotype prediction without requiring next-generation sequencing data.

## Data Availability

Haplo2D6 is accessible at https://bioinfo.dcc.ufmg.br/Haplo2D6/ without registration. Source code is available at https://github.com/LBS-UFMG/Haplo2D6.
